# The Effect of Fluticasone Nasal Spray on Patients with Chronic Rhinitis and Chronic Obstructive Pulmonary Disease

**DOI:** 10.3390/jcm14113637

**Published:** 2025-05-22

**Authors:** Zheng-Yan Dai, Yu-Ting Li, Jin-Yi Lin, Chien-Lin Liu, Yung-An Tsou, Chia-Der Lin, Chih-Jaan Tai, Liang-Chun Shih

**Affiliations:** 1 Department of Otorhinolaryngology-Head and Neck Surgery, China Medical University Hospital, Taichung 404327, Taiwan; 106001428@365.cmu.edu.tw (Z.-Y.D.); 027119@tool.caaumed.org.tw (Y.-T.L.); 032706@tool.caaumed.org.tw (J.-Y.L.); b0305043@cgu.edu.tw (C.-L.L.); 006638@tool.caaumed.org.tw (Y.-A.T.); 006355@tool.caaumed.org.tw (C.-D.L.); 003485@tool.caaumed.org.tw (C.-J.T.); 2 Department of Otorhinolaryngology-Head and Neck Surgery, Asia University Hospital, Taichung 404327, Taiwan; 3 School of Medicine, China Medical University, Taichung 404328, Taiwan

**Keywords:** chronic rhinitis, chronic obstructive pulmonary disease, nasal spray

## Abstract

**Background:** Although upper and lower respiratory tract diseases coexist, studies discussing the relationship between chronic rhinitis (CR) and chronic obstructive pulmonary disease (COPD) are limited. Fluticasone nasal sprays are common treatment options for patients with rhinitis. Therefore, we aimed to investigate the effects of fluticasone nasal spray on patients with both CR and COPD. **Methods**: A retrospective review was performed using data from former smokers with CR and COPD at China Medical University Hospital (CMUH). Based on their medication history, patients were allocated into Group A, who had received treatment with fluticasone nasal spray, and Group B, who had never received this treatment. Pulmonary function test results, including forced expiratory volume in 1 s (FEV1) and forced vital capacity (FVC), were collected for both groups before treatment and one year after treatment. Statistical analysis was performed to evaluate the impact of fluticasone nasal spray treatment on pulmonary function. **Results**: A total of 123 former smokers were included, with 62 patients in Group A and 61 patients in Group B. At baseline, there was no significant difference in age, sex, and pulmonary function between the two groups. After one year of treatment, Group A showed an upward trend in pulmonary function, with the FEV1 increasing from 1.613 ± 0.554 to 1.708 ± 0.675 (*p* < 0.05) and the FVC increasing from 2.540 ± 0.694 to 2.670 ± 0.839 (*p* < 0.05). On the other hand, Group B exhibited a downward trend in pulmonary function after one year, with the FEV1 decreasing from 1.609 ± 0.554 to 1.544 ± 0.517 (*p* < 0.05) and the FVC decreasing from 2.586 ± 0.665 to 2.495 ± 0.679 (*p* < 0.05). **Conclusions**: This retrospective study suggests that the combined use of fluticasone nasal spray may be associated with improved pulmonary function in former smokers with both CR and COPD. This finding supports the concept of “united airway disease”.

## 1. Introduction

Chronic rhinitis (CR) is characterized by inflammation of the mucous membranes in the nasal cavity, with symptoms such as rhinorrhea, nasal congestion, nasal itching, and sneezing that persist for more than 12 weeks [1]. Common treatment options for patients with rhinitis include antihistamines and corticosteroid nasal sprays.

Chronic obstructive pulmonary disease (COPD) is characterized by persistent airway inflammation, resulting in airflow obstruction that is difficult to reverse, impairing the movement of gases in and out of the respiratory tract. It includes two major types: chronic bronchitis and emphysema. Common symptoms include cough, sputum production, and wheezing. Smoking is the primary risk factor for COPD. Treatments include long-acting beta-agonists (LABAs), long-acting muscarinic antagonists (LAMAs), and inhaled corticosteroids (ICSs) [2].

Upper and lower respiratory tract diseases often coexist and are closely related. Moreover, treatments targeting upper respiratory tract diseases may improve the outcomes of lower respiratory tract diseases, which supports the concept of “united airway disease” [1]. The association between rhinitis and asthma has been extensively studied, and they have been found to be highly correlated. However, the relationship between rhinitis and COPD remains unclear.

From an epidemiological perspective, patients with COPD have a higher proportion of nasal symptoms than individuals without lung disease. A study conducted on 65 patients with moderate-to-severe COPD, using the Sinonasal Outcome Test (SNOT-20), revealed that 88% of the patients experienced nasal symptoms throughout the week, with nasal discharge being the most common symptom [3]. In another study conducted in London involving 61 patients with COPD, a high prevalence of nasal symptoms (75%), including nasal discharge (52.5%) and sneezing (45.9%), were observed [4]. Another large-scale study in Sweden (*n* = 8469) reported that 33% of participants had sinus symptoms. Approximately 40% of the patients with COPD exhibited notable nasal symptoms, which were more common among smokers [5].

In addition to nasal symptoms, patients with COPD have a higher incidence of chronic rhinosinusitis (CRS) compared to the general population. A study in the Netherlands assessed the prevalence of various comorbidities among 290 patients with COPD and 421 matched controls without lung disease. The CRS incidence in patients with COPD (12.4%) was significantly higher than that in the control group (2.5%) [6]. Another Canadian study with 73,364 participants demonstrated that CRS was more common among participants with a history of smoking, asthma, or COPD [7]. Furthermore, patients with CRS also have an increased incidence of lower respiratory tract diseases. A clinical evaluation study of 25 patients with CRS observed that 60% demonstrated related lower respiratory tract involvement, with 24% having asthma and 36% having small airway disease [8]. These studies indicate a strong association between CRS and COPD.

However, studies discussing the relationship between CR and COPD are limited. This is a novel research topic, as the impact of nasal treatment on COPD outcomes remains largely unexplored. Therefore, we aimed to study the effect of treatment targeting the nasal passages on COPD outcomes. We analyzed data from a group of patients with both CR and COPD and evaluated the effects of corticosteroid nasal spray.

## 2. Methods

### 2.1. Study Design

A retrospective review was conducted using data from former smokers with CR and COPD at China Medical University Hospital (CMUH), with all diagnoses confirmed by visiting staff. All patients received COPD treatment following the guidelines outlined by the Global Initiative for Chronic Obstructive Lung Disease (GOLD). This study protocol was approved by the ethics committee of CMUH, ensuring that all research procedures adhered to ethical standards and guidelines. The study was conducted under the ethical approval code CMUH111-REC3-139, and underscores our commitment to maintaining the highest ethical considerations in our research involving human participants.

We allocated the patients into two groups: (1) Group A, who had received treatment with fluticasone nasal spray; and (2) Group B, who had never received fluticasone nasal spray treatment. We evaluated whether the treatment for rhinitis impacted the effectiveness of COPD treatment. Pulmonary function test results, including forced expiratory volume in 1 s (FEV1) and forced vital capacity (FVC), were collected for both groups before treatment and one year after treatment. The initial fluticasone nasal spray dose was 110 mcg once daily (two sprays in each nostril). When symptoms were under control, the dose was reduced to 55 mcg once daily (one spray in each nostril).

### 2.2. Inclusion and Exclusion Criteria

Former smokers aged ≥18 years who were diagnosed with both CR and COPD based on clinical symptoms, medical history, and pulmonary function tests were included. According to the EPOS guidelines, the diagnosis of CR was determined based on symptoms such as rhinorrhea, nasal congestion, nasal itching, and sneezing, persisting for more than 12 weeks. According to the GOLD guidelines, the diagnosis of COPD was based on pulmonary function test results with a FEV1/FVC ratio < 70%.

Patients under 18 years of age, pregnant women, never-smokers, antihistamine users, and individuals with asthma, pneumonia, tuberculosis, cystic fibrosis, primary ciliary dyskinesia, lung cancer, lung transplant recipients, autoimmune diseases, genetic diseases, or other serious illnesses were excluded.

### 2.3. Data Collection

We reviewed the medical records of patients who visited CMUH between 2016 and 2023, and identified individuals diagnosed with CR and COPD. Patient history, clinical records, medication records, and pulmonary function test results were collected. We searched the clinical database and then manually checked the data to determine whether the participants met the inclusion and exclusion criteria.

### 2.4. Data Analysis

The data were analyzed using SPSS software (version 26, IBM, Armonk, NY, USA). We used an independent-sample *t*-test to compare groups A and B. A paired-sample *t*-test was used to evaluate the changes in pulmonary function within Group A and B over one year. The data did not significantly deviate from normality. Therefore, the *t*-test was appropriate for the analysis. Statistical significance was set at *p* < 0.05.

## 3. Results

In this study, 123 former smokers with CR and COPD were included. Group A comprised 62 patients who received treatment with fluticasone nasal spray, while Group B comprised 61 patients who had never received this treatment. The baseline characteristics of the patients are presented in Table 1. The values are expressed as the mean ± standard deviation (SD).

The average age of patients in Group A was 66.6 ± 11.0 years, comprising 84% men and 16% women. The average age of patients in Group B was 69.3 ± 10.0 years, comprising 82% men and 18% women. No significant difference in age, sex, and pulmonary function was observed between the two groups. Regression analysis revealed that there was no confounder, apart from fluticasone nasal spray, affecting the change in pulmonary function.

After one year of treatment, Group A revealed an upward trend in pulmonary function, with the FEV1 increasing from 1.613 ± 0.554 to 1.708 ± 0.675 (*p* < 0.05) and the FVC increasing from 2.540 ± 0.694 to 2.670 ± 0.839 (*p* < 0.05). Group B, which did not receive fluticasone nasal spray treatment, exhibited a downward trend in pulmonary function after one year, with the FEV1 decreasing from 1.609 ± 0.554 to 1.544 ± 0.517 (*p* < 0.05) and the FVC decreasing from 2.586 ± 0.665 to 2.495 ± 0.679 (*p* < 0.05). Detailed information is presented in Table 2.

## 4. Discussion

Smoking appears to be the cause of CR and COPD in these patients. Harmful components in cigarette smoke may act as irritants. Long-term cigarette smoke exposure may cause chronic inflammation of both the upper and lower airways. A close association between smoking and rhinitis has been reported. A study targeting the U.S. population observed that smoking was associated with an increased prevalence of CR. Smokers reported upper and lower respiratory tract diseases more frequently than non-smokers [9]. Another study that randomly sampled 2044 participants revealed that smoking was associated with an increased risk of non-infectious rhinitis (NIR) [10]. Furthermore, another study revealed that this relationship exhibited a dose–response pattern. A follow-up study involving 191 men found that smokers had a higher proportion of CR compared to non-smokers. A higher frequency of smoking was associated with an increased prevalence of CR among participants [11].

Nasal symptoms may exacerbate COPD. A survey of 4280 participants found that certain nasal symptoms and nasal symptom-provoking exposures, which are different from those typically associated with asthma, may indicate an increased risk of developing COPD [12]. Another study analyzing 2176 COPD patients found that comorbid CR is significantly associated with 30-day COPD-related readmissions [13]. Additionally, a study involving 107 COPD patients reported that a history of sinusitis is associated with treatment failure during acute exacerbations of COPD [14]. Furthermore, COPD also increases the risk of rhinitis. A study from Sweden reported that the risk factors for NIR include COPD, smoking, and allergies. The results suggest an association between NIR and inflammation of the upper and lower respiratory tracts in COPD [15].

The upper and lower airways share similar histology, are exposed to comparable irritants, and exhibit similar inflammatory responses. Upper and lower airways may influence each other through certain inflammatory mediators. If similar inflammatory responses could be found throughout the upper and lower airways, it would support the concept of “united airway disease”. Some studies have collected samples from upper and lower respiratory tracts and observed consistent inflammatory mediators. One study included 14 smokers with COPD, 7 smokers without COPD, and 7 controls, and examined their nasal and bronchial biopsy specimens. Squamous metaplasia occurs in the nasal and bronchial epithelium of smokers with or without COPD. Smokers with COPD exhibited a higher concentration of neutrophils in the nasal and bronchial mucosa, whereas smokers without COPD exhibited a higher concentration of eosinophils [16]. Another study involving 41 patients with COPD found that during exacerbations of COPD, there was a higher correlation between inflammation in the nasal cavity, sputum, and serum compared to stable states, with proportional increases in inflammatory mediators. The degree of upper respiratory tract inflammation correlates with that of the lower respiratory tract [17].

There have been two studies reporting contrasting results. One study included 47 patients with COPD and 12 healthy controls. Compared to the control group, patients with COPD exhibited elevated nasal and sputum IL-8 levels, indicating a correlation between the inflammation levels in the upper and lower respiratory tracts of patients with COPD [18]. The other study included 31 patients with COPD and 61 healthy controls. Patients with COPD reported increased nasal symptoms and nasal inflammation, with elevated levels of eotaxin, G-CSF, and IFN-γ in the nasal cavity. However, the IL-8 levels remained unchanged compared to the control group, which contrasts with the findings of the previous study [19].

The nasal patency may have influenced the pulmonary function test values. Group A, having received rhinitis treatment, had better nasal patency, resulting in better pulmonary function values. However, Group B, without rhinitis treatment, had poor nasal patency, resulting in worse pulmonary function values. Some studies are consistent with our findings. A study comprising 51 patients with COPD evaluated the airway using acoustic rhinometry and spirometry. COPD patients experienced chronic nasal symptoms, reduced nasal patency, and impaired pulmonary airflow, worsening the severity of COPD [20]. Another study included 42 patients with COPD and 12 healthy controls. Compared to the control group, the COPD group had a higher incidence of nasal symptoms and pathological findings from nasal endoscopy. The overall nasal airflow resistance was also higher in the COPD group than in the controls [21].

Both rhinitis treatment (corticosteroid nasal sprays) and COPD treatment (inhaled corticosteroids) contain fluticasone. Could these nasal sprays directly affect the lungs? A meta-analysis investigated the impact of corticosteroid nasal sprays on patients with both rhinitis and asthma. The study results indicated that patients who used corticosteroid nasal sprays had significant improvements in FEV1 compared to those who did not use the sprays. However, the improvement in FEV1 was most noticeable only when the patients were not receiving inhaled ICSs. Adding a corticosteroid nasal spray did not result in significant changes in FEV1 for patients who already used ICSs. This finding indicates that patients receiving ICSs have already achieved baseline control of their lower airway inflammation, thereby limiting the potential for further improvement through additional medications. This study can draw two possible conclusions: First, the improvement in FEV1 owing to corticosteroid nasal sprays may result from the sprays depositing in the lungs. Second, reducing inflammation in the upper airway through use of the corticosteroid nasal sprays may improve the lower airway outcomes [22].

In patients with COPD, using tiotropium (LAMA), salmeterol (LABA), or fluticasone (ICS) individually can slow down the rate of FEV1 decline [23,24]. Some studies have indicated that combining LAMAs, LABAs, and ICSs can achieve better results. A study indicated that the combined use of tiotropium, salmeterol, and fluticasone, compared to a placebo, improved pulmonary function and disease-specific quality of life, and reduced the number of hospitalizations for COPD exacerbations and all-cause hospitalizations. In contrast, compared to the placebo, the combined use of tiotropium and salmeterol did not statistically improve pulmonary function or hospitalization rates [25].

In another 52-week study, all COPD patients initially used triple therapy with tiotropium, salmeterol, and fluticasone. At the 12th week, patients were randomly assigned to either continue triple therapy or withdraw from fluticasone. The results showed that although pulmonary function declined in both groups, the decline was significantly greater in the fluticasone-withdrawal group compared to the fluticasone-continuation group [26].

Another systematic review indicated that most studies showed that COPD patients using ICS-containing medications had better pulmonary function compared to those using non-ICS-containing medications [27].

The differences in study design, ethnicity, and age may account for the variability in outcomes, where some studies report an improvement in pulmonary function whereas others show a decline, despite using the same medication. However, a consistent finding across studies is that COPD patients using ICSs exhibit better pulmonary function.

In this study, the corticosteroid nasal spray improved pulmonary function in patients with both rhinitis and COPD. Most patients with COPD primarily use LABAs and LAMAs, with fewer individuals using ICSs. In Group A, only three patients used ICSs, and in Group B, only two patients used ICSs. The improvement in pulmonary function from corticosteroid nasal sprays may present through two possible mechanisms. First, it could result from the deposition of the spray in the lungs. Second, reducing upper airway inflammation with a corticosteroid nasal spray may improve the lower airways. The low use of ICSs among our COPD patients may be due to the following reasons: According to the GOLD guidelines, LABAs or LAMAs are preferred for monotherapy, while ICS monotherapy is not recommended. For combination therapy, a LABA plus LAMA regimen is generally used.

Another similar study, focusing on patients with both rhinitis and COPD using corticosteroid nasal sprays, reported significant improvements in the COPD assessment test (CAT), the Sinonasal Outcome Test (SNOT-22), and modified Medical Research Council scores. This result indicates that corticosteroid nasal sprays have beneficial clinical effects on COPD symptoms and quality of life [28].

This study has several limitations. The sample size was relatively small, which may limit the statistical power of the findings. Additionally, the age and sex distributions between the two groups were not equivalent, potentially affecting the results. The study also evaluated changes over a short period of one year, leaving long-term effects unclear. Moreover, the focus on the Taiwanese population may limit the generalizability of the findings to other countries and ethnic groups. As this is a retrospective study, future large-scale, long-term prospective and randomized studies are needed to validate these results.

## 5. Conclusions

This retrospective study suggests that the combined use of fluticasone nasal spray may be associated with improved pulmonary function in former smokers with both CR and COPD. This finding supports the concept of “united airway disease”.

## Figures and Tables

**Table 1:** The baseline characteristics of the patients.

Characteristics	Group A	Group B	*p*-Value
	(*n* = 62)	(*n* = 61)	
Sex, *n* (%)			
Male	52 (84%)	50 (82%)	0.781
Female	10 (16%)	11 (18%)	
Age			
Mean ± SD	66.6 ± 11.0	69.3 ± 10.0	0.157
FEV1			
Mean ± SD	1.613 ± 0.554	1.609 ± 0.554	0.972
FVC			
Mean ± SD	2.540 ± 0.694	2.586 ± 0.665	0.709

Abbreviations: FEV1 = forced expiratory volume in 1 s; FVC = forced vital capacity.

**Table 2:** The pulmonary function test values of the patients.

Group	Pulmonary Function	Time		*p*-Value
		Before Treatment	One Year After Treatment	
Group A (*n* = 62)	FEV1			
	Mean ± SD	1.613 ± 0.554	1.708 ± 0.675	0.044 *
	FVC			
	Mean ± SD	2.540 ± 0.694	2.670 ± 0.839	0.022 *
Group B (*n* = 61)	FEV1			
	Mean ± SD	1.609 ± 0.554	1.544 ± 0.517	0.035 *
	FVC			
	Mean ± SD	2.586 ± 0.665	2.495 ± 0.679	0.034 *

Abbreviations: FEV1 = forced expiratory volume in 1 s; FVC = forced vital capacity. * *p* < 0.05.

## Data Availability

The datasets presented in this article are available from the corresponding author upon reasonable request.

## References

[1] Fokkens W.J., Lund V.J., Hopkins C., Hellings P.W., Kern R., Reitsma S., Toppila-Salmi S., Bernal-Sprekelsen M., Mullol J., Alobid I. (2020). European Position Paper on Rhinosinusitis and Nasal Polyps 2020. Rhinology.

[2] Agustí A., Celli B.R., Criner G.J., Halpin D., Anzueto A., Barnes P., Bourbeau J., Han M.K., Martinez F.J., de Oca M.M. (2023). Global Initiative for Chronic Obstructive Lung Disease 2023 Report: GOLD Executive Summary. Am. J. Respir. Crit. Care Med..

[3] Hurst J.R., Wilkinson T.M., Donaldson G.C., Wedzicha J.A. (2004). Upper airway symptoms and quality of life in chronic obstructive pulmonary disease (COPD). Respir. Med..

[4] Roberts N.J., Lloyd-Owen S.J., Rapado F., Patel I.S., Wilkinson T.M., Donaldson G.C., Wedzicha J.A. (2003). Relationship between chronic nasal and respiratory symptoms in patients with COPD. Respir. Med..

[5] Montnemery P., Svensson C., Ädelroth E., Löfdahl C.G., Andersson M., Greiff L., Persson C.G.A. (2001). Prevalence of nasal symptoms and their relation to self-reported asthma and chronic bronchitis/emphysema. Eur. Respir. J..

[6] van Manen J.G., Bindels P.J., Ijzermans C.J., van der Zee J.S., Bottema B.J., Schadé E. (2001). Prevalence of comorbidity in patients with a chronic airway obstruction and controls over the age of 40. J. Clin. Epidemiol..

[7] Chen Y., Dales R., Lin M. (2003). The epidemiology of chronic rhinosinusitis in Canadians. Laryngoscope.

[8] Ragab A., Clement P., Vincken W. (2004). Objective assessment of lower airway involvement in chronic rhinosinusitis. Am. J. Rhinol..

[9] Turkeltaub P.C., Gergen P.J. (1991). Prevalence of upper and lower respiratory conditions in the US population by social and environmental factors: Data from the second National Health and Nutrition Examination Survey, 1976 to 1980 (NHANES II). Ann. Allergy.

[10] Hellgren J., Lillienberg L., Jarlstedt J., Karlsson G., Torén K. (2002). Population-based study of non-infectious rhinitis in relation to occupational exposure, age, sex, and smoking. Am. J. Ind. Med..

[11] Annesi-Maesano I., Oryszczyn M.P., Neukirch F., Kauffmann F. (1997). Relationship of upper airway disease to tobacco smoking and allergic markers: A cohort study of men followed up for 5 years. Int. Arch. Allergy Immunol..

[12] Nihlén U., Montnémery P., Andersson M., Persson C.G., Nyberg P., Löfdahl C.G., Greiff L. (2008). Specific nasal symptoms and symptom-provoking factors may predict increased risk of developing COPD. Clin. Physiol. Funct. Imaging.

[13] Singh U., Wangia-Anderson V., Bernstein J.A. (2019). Chronic Rhinitis Is a High-Risk Comorbidity for 30-Day Hospital Readmission of Patients with Asthma and Chronic Obstructive Pulmonary Disease. J. Allergy Clin. Immunol. Pract..

[14] Dewan N.A., Rafique S., Kanwar B., Satpathy H., Ryschon K., Tillotson G.S., Niederman M.S. (2000). Acute exacerbation of COPD: Factors associated with poor treatment outcome. Chest.

[15] Bergqvist J., Andersson A., Olin A.C., Murgia N., Schiöler L., Bove M., Hellgren J. (2016). New evidence of increased risk of rhinitis in subjects with COPD: A longitudinal population study. Int. J. Chron. Obs. Pulm. Dis..

[16] Vachier I., Vignola A.M., Chiappara G., Bruno A., Meziane H., Godard P., Bousquet J., Chanez P. (2004). Inflammatory features of nasal mucosa in smokers with and without COPD. Thorax.

[17] Hurst J.R., Perera W.R., Wilkinson T.M., Donaldson G.C., Wedzicha J.A. (2006). Systemic and upper and lower airway inflammation at exacerbation of chronic obstructive pulmonary disease. Am. J. Respir. Crit. Care Med..

[18] Hurst J.R., Wilkinson T.M., Perera W.R., Donaldson G.C., Wedzicha J.A. (2005). Relationships among bacteria, upper airway, lower airway, and systemic inflammation in COPD. Chest.

[19] Hens G., Vanaudenaerde B.M., Bullens D.M.A., Piessens M., Decramer M., Dupont L.J., Ceuppens J.L., Hellings P.W. (2008). Sinonasal pathology in nonallergic asthma and COPD: “united airway disease” beyond the scope of allergy. Allergy.

[20] Hurst J.R., Kuchai R., Michael P., Perera W.R., Wilkinson T.M., Wedzicha J.A. (2006). Nasal symptoms, airway obstruction and disease severity in chronic obstructive pulmonary disease. Clin. Physiol. Funct. Imaging.

[21] Celakovsky P., Smatanova K., Kalfert D., Pracharova S., Koblizek V. (2015). Nasal symptomatology, obstruction, and paranasal sinus opacity in patients with chronic obstructive pulmonary disease. Acta Oto-Laryngol..

[22] Lohia S., Schlosser R.J., Soler Z.M. (2013). Impact of intranasal corticosteroids on asthma outcomes in allergic rhinitis: A meta-analysis. Allergy.

[23] Tashkin D.P., Celli B., Senn S., Burkhart D., Kesten S., Menjoge S., Decramer M. (2008). A 4-year trial of tiotropium in chronic obstructive pulmonary disease. N. Engl. J. Med..

[24] Calverley P.M., Anderson J.A., Celli B., Ferguson G.T., Jenkins C., Jones P.W., Yates J.C., Vestbo J. (2007). Salmeterol and fluticasone propionate and survival in chronic obstructive pulmonary disease. N. Engl. J. Med..

[25] Aaron S.D., Vandemheen K.L., Fergusson D., Maltais F., Bourbeau J., Goldstein R., Balter M., O’Donnell D., McIvor A., Sharma S. (2007). Tiotropium in combination with placebo, salmeterol, or fluticasone-salmeterol for treatment of chronic obstructive pulmonary disease: A randomized trial. Ann. Intern. Med..

[26] Magnussen H., Disse B., Rodriguez-Roisin R., Kirsten A., Watz H., Tetzlaff K., Towse L., Finnigan H., Dahl R., Decramer M. (2014). Withdrawal of inhaled glucocorticoids and exacerbations of COPD. N. Engl. J. Med..

[27] Whittaker H.R., Jarvis D., Sheikh M.R., Kiddle S.J., Quint J.K. (2019). Inhaled corticosteroids and FEV1 decline in chronic obstructive pulmonary disease: A systematic review. Respir. Res..

[28] Calabrese C., Costigliola A., Maffei M., Simeon V., Perna F., Tremante E., Merola E., Leone C.A., Bianco A. (2018). Clinical impact of nasal budesonide treatment on COPD patients with coexistent rhinitis. Int. J. Chronic Obs. Pulm. Dis..

